# Bis(2,2′-bipyridyl-κ^2^ *N*,*N*′)(nitrato-κ^2^ *O*,*O*′)(trifluoro­acetato-κ*O*)cadmium(II)

**DOI:** 10.1107/S1600536809016717

**Published:** 2009-05-14

**Authors:** Wenzeng Duan, Junshan Sun, Yudao Ma, Rentao Wu

**Affiliations:** a Department of Chemistry and Environmental Science, Taishan University, 271021 Taian, Shandong, People’s Republic of China; b School of Chemistry and Chemical Engineering, Shandong University, Jinan, 250100, People’s Republic of China; c Department of Materials and Chemical Engineering, Taishan University, 271021 Taian, Shandong, People’s Republic of China

## Abstract

In the title complex, [Cd(C_2_F_3_O_2_)(NO_3_)(C_10_H_8_N_2_)_2_], the Cd(II) ion is hepta­coordinated by two chelating 2,2′-bipyridyl ligands [Cd⋯N 2.370 (6)–2.416 (6) Å], one carboxyl­ate O atom [Cd⋯O 2.290 (6) Å] from the trifluoro­acetate ligand and two O atoms [Cd⋯O 2.386 (6), 2.633 (6) Å] from a chelating nitrate anion. The trifluoro­methyl fragment is rotationally disordered between two orientations in a 0.640 (7):0.360 (7) ratio. In the crystal, weak inter­molecular C—H⋯O hydrogen bonds contribute to the crystal packing stability.

## Related literature

For the crystal structures of related compounds with nickel, see: Eremenko *et al.* (1999 ▶); Rajaraman *et al.* (2005 ▶).
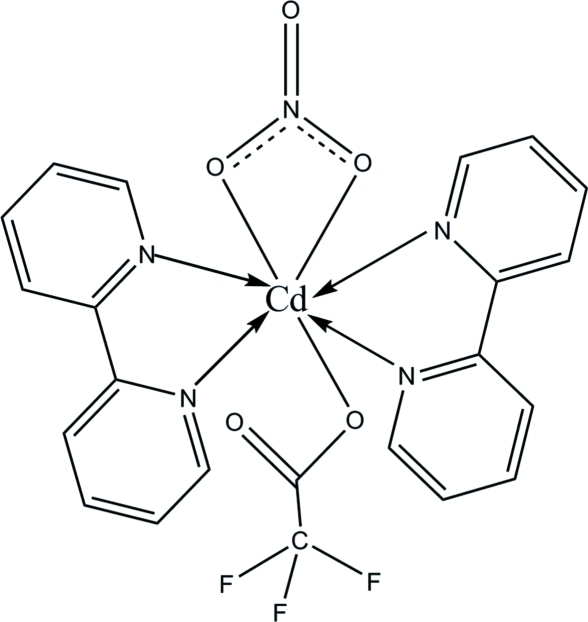


## Experimental

### 

#### Crystal data

[Cd(C_2_F_3_O_2_)(NO_3_)(C_10_H_8_N_2_)_2_]
*M* *_r_* = 599.80
Monoclinic, 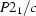

*a* = 14.9327 (13) Å
*b* = 9.6613 (8) Å
*c* = 15.9859 (14) Å
β = 93.568 (2)°
*V* = 2301.8 (3) Å^3^
*Z* = 4
Mo *K*α radiation
μ = 1.02 mm^−1^
*T* = 273 K
0.12 × 0.10 × 0.06 mm

#### Data collection

Bruker Smart APEX diffractometer
Absorption correction: multi-scan (*SADABS*; Sheldrick, 1996 ▶) *T*_min_ = 0.888, *T*_max_ = 0.941
11743 measured reflections
4075 independent reflections
3128 reflections with *I* > 2σ(*I*)
*R*_int_ = 0.029

#### Refinement

*R*[*F* ^2^ > 2σ(*F* ^2^)] = 0.059
*wR*(*F* ^2^) = 0.170
*S* = 1.02
4075 reflections
329 parameters
516 restraints
H-atom parameters constrained
Δρ_max_ = 0.94 e Å^−3^
Δρ_min_ = −1.42 e Å^−3^

### 

Data collection: *SMART* (Siemens, 1996 ▶); cell refinement: *SAINT* (Siemens, 1996 ▶); data reduction: *SAINT*; program(s) used to solve structure: *SHELXS97* (Sheldrick, 2008 ▶); program(s) used to refine structure: *SHELXL97* (Sheldrick, 2008 ▶); molecular graphics: *SHELXTL* (Sheldrick, 2008 ▶); software used to prepare material for publication: *SHELXTL*.

## Supplementary Material

Crystal structure: contains datablocks I, global. DOI: 10.1107/S1600536809016717/cv2556sup1.cif

Structure factors: contains datablocks I. DOI: 10.1107/S1600536809016717/cv2556Isup2.hkl

Additional supplementary materials:  crystallographic information; 3D view; checkCIF report

## Figures and Tables

**Table 1:** Hydrogen-bond geometry (Å, °)

*D*—H⋯*A*	*D*—H	H⋯*A*	*D*⋯*A*	*D*—H⋯*A*
C7—H7⋯O5^i^	0.93	2.44	3.160 (13)	134
C19—H19⋯O2^ii^	0.93	2.52	3.320 (11)	145
C13—H13⋯O3^iii^	0.93	2.43	3.287 (12)	152
C14—H14⋯O2^iv^	0.93	2.44	3.294 (11)	152

Symmetry codes: (i) 
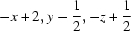
; (ii) 
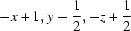
; (iii) 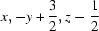; (iv) 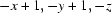.

**Fig. 1.**
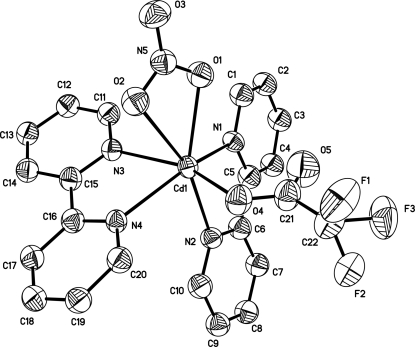
The molecular structure of (I) showing the atomic labeling and 30% probability displacement ellipsoids for non-H atoms. Only major part of the disordered trifluoromethyl group is shown. H atoms omitted for clarity.

## supplementary crystallographic information

### Comment

In recent years, researchers showed considerable interest in the physical and chemical properties of mono- and polynuclear complexes of transition metals. especially in the metal complexes with carboxylates, which are among the most investigated complexes in the field of coordination chemistry. Due to their versatile bonding modes with metal ions, they have also been used in the synthesis of mononuclear (Eremenko, *et al.*, 1999) and multi-nuclear (Rajaraman, *et al.*, 2005) compounds. Herein, we report the crystal structure of the title compound, (I).

In (I) (Fig. 1), the Cd^II^ ion is seven-coordinated by four N and three O atoms. Weak intermolecular C—H···O hydrogen bonds (Table 1) stabilize the crystal packing.

### Experimental

A mixture of trifluoroacetate(1 mmol), 2, 2'-bipyridine(bpy)(1 mmol), cadmium nitrate tetrahydrate (0.5 mmol), NaOH(1 mmol) and H2O(15 ml) were placed in a Teflon-lined stainless steel vessel, and heated to 418 K for 48 h. It was then cooled to room temperature over a period of 24 h. Colourless crystals suitable for X-ray diffraction analysis were obtained.

### Refinement

All H atoms were positioned geometrically with C—H = 0.93 Å are refined as riding model withh *U*_iso_(H) = 1.2 times *U*_eq_(C). Trifluoromethyl fragment was treated as rotationally disordered between two orientations with the refined occupancies of  0.640 (7) and 0.360 (7), respectively.

### Crystal data

**Table:**

[Cd(C_2_F_3_O_2_)(NO_3_)(C_10_H_8_N_2_)_2_]	*F*(000) = 1192
*M**_r_* = 599.80	*D*_x_ = 1.731 Mg m^−^^3^
Monoclinic, *P*2_1_/*c*	Mo *K*α radiation, λ = 0.71073 Å
*a* = 14.9327 (13) Å	Cell parameters from 3157 reflections
*b* = 9.6613 (8) Å	θ = 2.5–23.1°
*c* = 15.9859 (14) Å	µ = 1.02 mm^−^^1^
β = 93.568 (2)°	*T* = 273 K
*V* = 2301.8 (3) Å^3^	Block, colourless
*Z* = 4	0.12 × 0.10 × 0.06 mm

### Data collection

**Table:**

Bruker Smart APEX diffractometer	4075 independent reflections
Radiation source: fine-focus sealed tube	3128 reflections with *I* > 2σ(*I*)
graphite	*R*_int_ = 0.029
phi and ω scans	θ_max_ = 25.1°, θ_min_ = 1.4°
Absorption correction: multi-scan (*SADABS*; Sheldrick, 1996)	*h* = −17→17
*T*_min_ = 0.888, *T*_max_ = 0.941	*k* = −11→8
11743 measured reflections	*l* = −18→19

### Refinement

**Table:**

Refinement on *F*^2^	Primary atom site location: structure-invariant direct methods
Least-squares matrix: full	Secondary atom site location: difference Fourier map
*R*[*F*^2^ > 2σ(*F*^2^)] = 0.059	Hydrogen site location: inferred from neighbouring sites
*wR*(*F*^2^) = 0.170	H-atom parameters constrained
*S* = 1.02	*w* = 1/[σ^2^(*F*_o_^2^) + (0.080*P*)^2^ + 9.6055*P*] where *P* = (*F*_o_^2^ + 2*F*_c_^2^)/3
4075 reflections	(Δ/σ)_max_ = 0.006
329 parameters	Δρ_max_ = 0.94 e Å^−^^3^
516 restraints	Δρ_min_ = −1.42 e Å^−^^3^

### Special details

**Table:**

Geometry. All e.s.d.'s (except the e.s.d. in the dihedral angle between two l.s. planes) are estimated using the full covariance matrix. The cell e.s.d.'s are taken into account individually in the estimation of e.s.d.'s in distances, angles and torsion angles; correlations between e.s.d.'s in cell parameters are only used when they are defined by crystal symmetry. An approximate (isotropic) treatment of cell e.s.d.'s is used for estimating e.s.d.'s involving l.s. planes.
Refinement. Refinement of *F*^2^ against ALL reflections. The weighted *R*-factor *wR* and goodness of fit *S* are based on *F*^2^, conventional *R*-factors *R* are based on *F*, with *F* set to zero for negative *F*^2^. The threshold expression of *F*^2^ > σ(*F*^2^) is used only for calculating *R*-factors(gt) *etc*. and is not relevant to the choice of reflections for refinement. *R*-factors based on *F*^2^ are statistically about twice as large as those based on *F*, and *R*- factors based on ALL data will be even larger.

### Fractional atomic coordinates and isotropic or equivalent isotropic displacement parameters (Å^2^)

**Table:**

	*x*	*y*	*z*	*U*_iso_*/*U*_eq_	Occ. (<1)
Cd1	0.73713 (3)	0.51539 (5)	0.16084 (3)	0.0502 (2)	
O1	0.7323 (4)	0.7621 (7)	0.1656 (4)	0.0763 (10)	
O2	0.6002 (4)	0.6880 (7)	0.1509 (4)	0.0773 (11)	
O3	0.6236 (5)	0.9068 (7)	0.1476 (4)	0.0931 (18)	
O4	0.7112 (5)	0.4828 (7)	0.2991 (4)	0.0792 (10)	
O5	0.8383 (6)	0.5634 (11)	0.3232 (6)	0.128 (2)	
N1	0.8898 (4)	0.5660 (7)	0.1289 (4)	0.0559 (15)	
N2	0.8265 (4)	0.3090 (7)	0.1600 (4)	0.0577 (16)	
N3	0.6969 (4)	0.5131 (6)	0.0152 (4)	0.0527 (15)	
N4	0.6102 (4)	0.3705 (6)	0.1314 (4)	0.0521 (14)	
N5	0.6504 (5)	0.7896 (8)	0.1543 (4)	0.0679 (14)	
F1	0.6917 (8)	0.4254 (18)	0.4519 (9)	0.1036 (14)	0.428 (7)
F2	0.8262 (10)	0.3799 (16)	0.4695 (9)	0.1036 (14)	0.428 (7)
F3	0.7842 (17)	0.580 (2)	0.4859 (17)	0.206 (12)	0.428 (7)
F1'	0.7171 (8)	0.5430 (14)	0.4766 (6)	0.113 (5)	0.572 (7)
F2'	0.7697 (8)	0.3425 (11)	0.4502 (7)	0.1036 (14)	0.572 (7)
F3'	0.8551 (7)	0.5117 (12)	0.4745 (7)	0.1036 (14)	0.572 (7)
C1	0.9188 (6)	0.6926 (10)	0.1099 (6)	0.0709 (12)	
H1	0.8782	0.7656	0.1092	0.085*	
C2	1.0062 (6)	0.7201 (10)	0.0911 (6)	0.0740 (15)	
H2	1.0241	0.8094	0.0781	0.089*	
C3	1.0650 (6)	0.6135 (10)	0.0922 (6)	0.0743 (14)	
H3	1.1243	0.6284	0.0799	0.089*	
C4	1.0364 (6)	0.4838 (9)	0.1116 (6)	0.0720 (13)	
H4	1.0765	0.4101	0.1123	0.086*	
C5	0.9488 (6)	0.4609 (9)	0.1302 (6)	0.0689 (12)	
C6	0.9155 (6)	0.3222 (9)	0.1502 (6)	0.0675 (12)	
C7	0.9715 (6)	0.2077 (9)	0.1592 (6)	0.0709 (13)	
H7	1.0328	0.2173	0.1532	0.085*	
C8	0.9367 (6)	0.0811 (10)	0.1766 (6)	0.0725 (14)	
H8	0.9741	0.0043	0.1830	0.087*	
C9	0.8474 (6)	0.0680 (10)	0.1846 (6)	0.0713 (14)	
H9	0.8222	−0.0175	0.1962	0.086*	
C10	0.7951 (6)	0.1839 (9)	0.1752 (6)	0.0710 (12)	
H10	0.7336	0.1742	0.1797	0.085*	
C11	0.7339 (6)	0.5950 (9)	−0.0407 (5)	0.0667 (12)	
H11	0.7785	0.6563	−0.0213	0.080*	
C12	0.7101 (6)	0.5938 (9)	−0.1246 (5)	0.0678 (14)	
H12	0.7378	0.6528	−0.1610	0.081*	
C13	0.6456 (6)	0.5054 (9)	−0.1536 (6)	0.0688 (13)	
H13	0.6290	0.5008	−0.2106	0.083*	
C14	0.6042 (6)	0.4213 (9)	−0.0969 (5)	0.0684 (13)	
H14	0.5582	0.3619	−0.1154	0.082*	
C15	0.6322 (6)	0.4267 (9)	−0.0128 (5)	0.0642 (11)	
C16	0.5884 (5)	0.3408 (9)	0.0510 (5)	0.0644 (11)	
C17	0.5253 (6)	0.2399 (9)	0.0290 (6)	0.0680 (13)	
H17	0.5104	0.2206	−0.0271	0.082*	
C18	0.4850 (6)	0.1688 (9)	0.0905 (6)	0.0698 (13)	
H18	0.4442	0.0987	0.0763	0.084*	
C19	0.5047 (5)	0.2006 (9)	0.1722 (6)	0.0679 (14)	
H19	0.4765	0.1552	0.2146	0.082*	
C20	0.5677 (5)	0.3019 (9)	0.1906 (6)	0.0669 (12)	
H20	0.5815	0.3241	0.2466	0.080*	
C21	0.7753 (7)	0.5073 (11)	0.3446 (6)	0.0822 (16)	
C22	0.7743 (6)	0.4755 (11)	0.4334 (8)	0.1036 (14)	

### Atomic displacement parameters (Å^2^)

**Table:**

	*U*^11^	*U*^22^	*U*^33^	*U*^12^	*U*^13^	*U*^23^
Cd1	0.0439 (3)	0.0563 (4)	0.0505 (3)	−0.0040 (2)	0.0033 (2)	−0.0030 (2)
O1	0.070 (2)	0.0743 (17)	0.085 (2)	−0.002 (2)	0.005 (2)	−0.003 (2)
O2	0.068 (2)	0.081 (2)	0.083 (2)	0.0039 (18)	0.006 (2)	−0.004 (2)
O3	0.105 (4)	0.079 (4)	0.094 (4)	0.028 (3)	0.004 (3)	−0.006 (3)
O4	0.078 (2)	0.098 (2)	0.0612 (14)	−0.006 (2)	0.0060 (19)	0.004 (2)
O5	0.114 (4)	0.154 (4)	0.116 (4)	−0.029 (4)	0.009 (4)	0.011 (4)
N1	0.051 (4)	0.054 (4)	0.063 (4)	−0.006 (3)	0.008 (3)	−0.003 (3)
N2	0.052 (4)	0.056 (4)	0.065 (4)	−0.002 (3)	0.003 (3)	0.003 (3)
N3	0.048 (3)	0.059 (4)	0.051 (3)	−0.001 (3)	0.006 (3)	0.001 (3)
N4	0.048 (3)	0.059 (4)	0.050 (3)	−0.005 (3)	0.003 (3)	0.003 (3)
N5	0.068 (3)	0.067 (3)	0.069 (3)	0.004 (3)	0.008 (3)	−0.008 (3)
F1	0.100 (3)	0.122 (3)	0.087 (3)	0.000 (3)	−0.005 (3)	0.010 (3)
F2	0.100 (3)	0.122 (3)	0.087 (3)	0.000 (3)	−0.005 (3)	0.010 (3)
F3	0.207 (12)	0.206 (12)	0.206 (12)	0.0001 (11)	0.0128 (13)	−0.0002 (11)
F1'	0.115 (9)	0.166 (12)	0.061 (6)	0.071 (9)	0.029 (6)	−0.019 (6)
F2'	0.100 (3)	0.122 (3)	0.087 (3)	0.000 (3)	−0.005 (3)	0.010 (3)
F3'	0.100 (3)	0.122 (3)	0.087 (3)	0.000 (3)	−0.005 (3)	0.010 (3)
C1	0.062 (2)	0.072 (2)	0.080 (2)	−0.005 (2)	0.009 (2)	−0.001 (2)
C2	0.064 (3)	0.075 (3)	0.084 (3)	−0.009 (3)	0.011 (3)	−0.001 (3)
C3	0.062 (3)	0.078 (3)	0.084 (3)	−0.006 (2)	0.011 (2)	−0.002 (3)
C4	0.060 (2)	0.075 (3)	0.082 (3)	−0.004 (2)	0.009 (2)	−0.002 (2)
C5	0.059 (2)	0.073 (2)	0.075 (2)	−0.005 (2)	0.008 (2)	−0.001 (2)
C6	0.060 (2)	0.069 (2)	0.074 (2)	−0.002 (2)	0.005 (2)	0.000 (2)
C7	0.062 (2)	0.073 (3)	0.078 (3)	0.000 (2)	0.007 (2)	0.000 (2)
C8	0.067 (3)	0.072 (3)	0.079 (3)	0.002 (2)	0.005 (2)	0.000 (3)
C9	0.067 (3)	0.070 (3)	0.078 (3)	−0.001 (3)	0.005 (3)	0.001 (3)
C10	0.064 (2)	0.074 (2)	0.075 (2)	−0.003 (2)	0.006 (2)	0.003 (2)
C11	0.060 (2)	0.071 (2)	0.070 (2)	−0.003 (2)	0.006 (2)	0.003 (2)
C12	0.064 (3)	0.073 (3)	0.067 (3)	0.001 (3)	0.008 (3)	0.006 (3)
C13	0.066 (3)	0.075 (3)	0.066 (3)	0.000 (2)	0.002 (2)	0.003 (2)
C14	0.064 (2)	0.073 (3)	0.068 (2)	−0.001 (2)	0.001 (2)	0.002 (2)
C15	0.058 (2)	0.069 (2)	0.066 (2)	−0.0006 (19)	0.0032 (19)	0.000 (2)
C16	0.056 (2)	0.069 (2)	0.068 (2)	−0.0015 (19)	0.0023 (19)	0.002 (2)
C17	0.061 (2)	0.072 (3)	0.071 (2)	−0.005 (2)	0.001 (2)	0.002 (2)
C18	0.061 (3)	0.072 (3)	0.076 (3)	−0.008 (2)	0.002 (2)	0.003 (2)
C19	0.058 (3)	0.071 (3)	0.075 (3)	−0.004 (2)	0.007 (3)	0.008 (3)
C20	0.060 (2)	0.073 (2)	0.068 (2)	−0.004 (2)	0.005 (2)	0.004 (2)
C21	0.073 (3)	0.099 (3)	0.074 (3)	−0.010 (3)	0.002 (3)	0.005 (3)
C22	0.100 (3)	0.122 (3)	0.087 (3)	0.000 (3)	−0.005 (3)	0.010 (3)

### Geometric parameters (Å, °)

**Table:**

Cd1—O4	2.289 (6)	C3—H3	0.9300
Cd1—N3	2.367 (6)	C4—C5	1.377 (12)
Cd1—N4	2.379 (6)	C4—H4	0.9300
Cd1—O1	2.386 (6)	C5—C6	1.471 (12)
Cd1—N2	2.400 (6)	C6—C7	1.389 (12)
Cd1—N1	2.417 (6)	C7—C8	1.365 (12)
Cd1—O2	2.636 (6)	C7—H7	0.9300
O1—N5	1.254 (8)	C8—C9	1.354 (12)
O2—N5	1.235 (9)	C8—H8	0.9300
O3—N5	1.203 (9)	C9—C10	1.368 (12)
O4—C21	1.190 (11)	C9—H9	0.9300
O5—C21	1.156 (12)	C10—H10	0.9300
N1—C1	1.339 (11)	C11—C12	1.366 (11)
N1—C5	1.343 (11)	C11—H11	0.9300
N2—C10	1.325 (10)	C12—C13	1.347 (12)
N2—C6	1.354 (10)	C12—H12	0.9300
N3—C15	1.334 (10)	C13—C14	1.389 (12)
N3—C11	1.339 (10)	C13—H13	0.9300
N4—C16	1.336 (10)	C14—C15	1.383 (11)
N4—C20	1.347 (10)	C14—H14	0.9300
F1—C22	1.374 (13)	C15—C16	1.497 (11)
F2—C22	1.316 (13)	C16—C17	1.386 (11)
F3—C22	1.316 (15)	C17—C18	1.369 (12)
F1'—C22	1.306 (11)	C17—H17	0.9300
F2'—C22	1.315 (12)	C18—C19	1.355 (12)
F3'—C22	1.382 (12)	C18—H18	0.9300
C1—C2	1.384 (12)	C19—C20	1.377 (11)
C1—H1	0.9300	C19—H19	0.9300
C2—C3	1.352 (12)	C20—H20	0.9300
C2—H2	0.9300	C21—C22	1.454 (15)
C3—C4	1.366 (12)		
			
O4—Cd1—N3	154.2 (2)	C7—C8—H8	120.2
O4—Cd1—N4	85.9 (2)	C8—C9—C10	118.1 (9)
N3—Cd1—N4	69.5 (2)	C8—C9—H9	120.9
O4—Cd1—O1	95.7 (2)	C10—C9—H9	120.9
N3—Cd1—O1	92.0 (2)	N2—C10—C9	124.1 (8)
N4—Cd1—O1	124.7 (2)	N2—C10—H10	117.9
O4—Cd1—N2	91.1 (2)	C9—C10—H10	117.9
N3—Cd1—N2	95.4 (2)	N3—C11—C12	123.9 (8)
N4—Cd1—N2	86.9 (2)	N3—C11—H11	118.1
O1—Cd1—N2	148.0 (2)	C12—C11—H11	118.1
O4—Cd1—N1	116.8 (2)	C13—C12—C11	118.7 (9)
N3—Cd1—N1	88.8 (2)	C13—C12—H12	120.6
N4—Cd1—N1	145.8 (2)	C11—C12—H12	120.6
O1—Cd1—N1	80.6 (2)	C12—C13—C14	118.9 (9)
N2—Cd1—N1	68.6 (2)	C12—C13—H13	120.6
O4—Cd1—O2	88.2 (2)	C14—C13—H13	120.6
N3—Cd1—O2	78.3 (2)	C15—C14—C13	119.5 (8)
N4—Cd1—O2	75.8 (2)	C15—C14—H14	120.3
O1—Cd1—O2	49.1 (2)	C13—C14—H14	120.3
N2—Cd1—O2	162.7 (2)	N3—C15—C14	121.3 (8)
N1—Cd1—O2	126.7 (2)	N3—C15—C16	117.1 (7)
N5—O1—Cd1	103.8 (5)	C14—C15—C16	121.6 (8)
N5—O2—Cd1	91.9 (5)	N4—C16—C17	121.1 (8)
C21—O4—Cd1	112.6 (7)	N4—C16—C15	116.5 (7)
C1—N1—C5	118.2 (7)	C17—C16—C15	122.4 (8)
C1—N1—Cd1	123.7 (5)	C18—C17—C16	119.5 (8)
C5—N1—Cd1	118.1 (5)	C18—C17—H17	120.2
C10—N2—C6	117.9 (7)	C16—C17—H17	120.2
C10—N2—Cd1	123.6 (6)	C19—C18—C17	120.0 (8)
C6—N2—Cd1	118.2 (5)	C19—C18—H18	120.0
C15—N3—C11	117.7 (7)	C17—C18—H18	120.0
C15—N3—Cd1	118.1 (5)	C18—C19—C20	118.1 (8)
C11—N3—Cd1	124.1 (5)	C18—C19—H19	120.9
C16—N4—C20	118.2 (7)	C20—C19—H19	120.9
C16—N4—Cd1	117.4 (5)	N4—C20—C19	123.0 (8)
C20—N4—Cd1	123.7 (5)	N4—C20—H20	118.5
O3—N5—O2	123.2 (8)	C19—C20—H20	118.5
O3—N5—O1	121.9 (8)	O5—C21—O4	123.5 (11)
O2—N5—O1	114.9 (7)	O5—C21—C22	116.5 (10)
N1—C1—C2	123.2 (9)	O4—C21—C22	119.9 (10)
N1—C1—H1	118.4	F1'—C22—F2'	109.6 (11)
C2—C1—H1	118.4	F1'—C22—F3	48.0 (11)
C3—C2—C1	118.1 (9)	F2'—C22—F3	128.6 (18)
C3—C2—H2	120.9	F1'—C22—F2	120.3 (13)
C1—C2—H2	120.9	F2'—C22—F2	42.4 (7)
C2—C3—C4	119.3 (9)	F3—C22—F2	102.6 (14)
C2—C3—H3	120.4	F1'—C22—F1	55.7 (9)
C4—C3—H3	120.4	F2'—C22—F1	63.4 (9)
C3—C4—C5	120.8 (9)	F3—C22—F1	101.5 (13)
C3—C4—H4	119.6	F2—C22—F1	99.8 (11)
C5—C4—H4	119.6	F1'—C22—F3'	101.7 (10)
N1—C5—C4	120.4 (8)	F2'—C22—F3'	101.9 (9)
N1—C5—C6	117.5 (7)	F3—C22—F3'	56.1 (10)
C4—C5—C6	122.1 (8)	F2—C22—F3'	59.7 (8)
N2—C6—C7	120.1 (8)	F1—C22—F3'	139.1 (13)
N2—C6—C5	117.3 (8)	F1'—C22—C21	117.3 (10)
C7—C6—C5	122.6 (8)	F2'—C22—C21	114.2 (10)
C8—C7—C6	120.0 (8)	F3—C22—C21	117.0 (17)
C8—C7—H7	120.0	F2—C22—C21	122.4 (11)
C6—C7—H7	120.0	F1—C22—C21	110.4 (10)
C9—C8—C7	119.6 (9)	F3'—C22—C21	110.4 (10)
C9—C8—H8	120.2		
			
O4—Cd1—O1—N5	−85.4 (5)	Cd1—O1—N5—O3	−175.2 (7)
N3—Cd1—O1—N5	69.9 (5)	Cd1—O1—N5—O2	5.0 (8)
N4—Cd1—O1—N5	3.6 (6)	C5—N1—C1—C2	0.3 (13)
N2—Cd1—O1—N5	173.4 (5)	Cd1—N1—C1—C2	−179.5 (7)
N1—Cd1—O1—N5	158.4 (5)	N1—C1—C2—C3	−0.1 (14)
O2—Cd1—O1—N5	−2.8 (4)	C1—C2—C3—C4	0.0 (14)
O4—Cd1—O2—N5	101.9 (5)	C2—C3—C4—C5	−0.2 (15)
N3—Cd1—O2—N5	−100.2 (5)	C1—N1—C5—C4	−0.6 (13)
N4—Cd1—O2—N5	−171.8 (5)	Cd1—N1—C5—C4	179.3 (7)
O1—Cd1—O2—N5	2.8 (4)	C1—N1—C5—C6	−179.3 (8)
N2—Cd1—O2—N5	−170.4 (6)	Cd1—N1—C5—C6	0.6 (10)
N1—Cd1—O2—N5	−20.6 (6)	C3—C4—C5—N1	0.5 (14)
N3—Cd1—O4—C21	178.0 (7)	C3—C4—C5—C6	179.1 (9)
N4—Cd1—O4—C21	160.1 (7)	C10—N2—C6—C7	−2.4 (12)
O1—Cd1—O4—C21	−75.4 (7)	Cd1—N2—C6—C7	172.5 (6)
N2—Cd1—O4—C21	73.3 (7)	C10—N2—C6—C5	177.7 (8)
N1—Cd1—O4—C21	6.9 (8)	Cd1—N2—C6—C5	−7.5 (10)
O2—Cd1—O4—C21	−123.9 (7)	N1—C5—C6—N2	4.6 (12)
O4—Cd1—N1—C1	−103.4 (7)	C4—C5—C6—N2	−174.1 (8)
N3—Cd1—N1—C1	80.5 (7)	N1—C5—C6—C7	−175.4 (8)
N4—Cd1—N1—C1	129.6 (6)	C4—C5—C6—C7	5.9 (14)
O1—Cd1—N1—C1	−11.7 (6)	N2—C6—C7—C8	1.0 (13)
N2—Cd1—N1—C1	176.8 (7)	C5—C6—C7—C8	−179.1 (9)
O2—Cd1—N1—C1	6.0 (7)	C6—C7—C8—C9	0.4 (14)
O4—Cd1—N1—C5	76.8 (6)	C7—C8—C9—C10	−0.4 (14)
N3—Cd1—N1—C5	−99.4 (6)	C6—N2—C10—C9	2.5 (13)
N4—Cd1—N1—C5	−50.2 (8)	Cd1—N2—C10—C9	−172.0 (7)
O1—Cd1—N1—C5	168.4 (6)	C8—C9—C10—N2	−1.1 (14)
N2—Cd1—N1—C5	−3.1 (6)	C15—N3—C11—C12	−0.9 (12)
O2—Cd1—N1—C5	−173.9 (6)	Cd1—N3—C11—C12	−179.6 (6)
O4—Cd1—N2—C10	61.6 (7)	N3—C11—C12—C13	0.0 (14)
N3—Cd1—N2—C10	−93.3 (7)	C11—C12—C13—C14	1.6 (13)
N4—Cd1—N2—C10	−24.3 (7)	C12—C13—C14—C15	−2.2 (13)
O1—Cd1—N2—C10	164.1 (6)	C11—N3—C15—C14	0.2 (12)
N1—Cd1—N2—C10	−179.9 (7)	Cd1—N3—C15—C14	179.0 (6)
O2—Cd1—N2—C10	−25.6 (11)	C11—N3—C15—C16	−177.1 (7)
O4—Cd1—N2—C6	−112.9 (6)	Cd1—N3—C15—C16	1.7 (10)
N3—Cd1—N2—C6	92.2 (6)	C13—C14—C15—N3	1.3 (13)
N4—Cd1—N2—C6	161.2 (6)	C13—C14—C15—C16	178.5 (8)
O1—Cd1—N2—C6	−10.4 (8)	C20—N4—C16—C17	−1.6 (12)
N1—Cd1—N2—C6	5.6 (6)	Cd1—N4—C16—C17	169.2 (6)
O2—Cd1—N2—C6	159.9 (7)	C20—N4—C16—C15	175.6 (7)
O4—Cd1—N3—C15	−25.2 (9)	Cd1—N4—C16—C15	−13.6 (9)
N4—Cd1—N3—C15	−6.1 (6)	N3—C15—C16—N4	8.0 (11)
O1—Cd1—N3—C15	−132.6 (6)	C14—C15—C16—N4	−169.3 (8)
N2—Cd1—N3—C15	78.6 (6)	N3—C15—C16—C17	−174.9 (8)
N1—Cd1—N3—C15	146.9 (6)	C14—C15—C16—C17	7.8 (13)
O2—Cd1—N3—C15	−85.2 (6)	N4—C16—C17—C18	−0.4 (13)
O4—Cd1—N3—C11	153.5 (6)	C15—C16—C17—C18	−177.3 (8)
N4—Cd1—N3—C11	172.6 (7)	C16—C17—C18—C19	2.2 (14)
O1—Cd1—N3—C11	46.1 (6)	C17—C18—C19—C20	−2.1 (13)
N2—Cd1—N3—C11	−102.7 (6)	C16—N4—C20—C19	1.8 (12)
N1—Cd1—N3—C11	−34.4 (6)	Cd1—N4—C20—C19	−168.5 (6)
O2—Cd1—N3—C11	93.6 (6)	C18—C19—C20—N4	0.1 (13)
O4—Cd1—N4—C16	−177.7 (6)	Cd1—O4—C21—O5	12.3 (15)
N3—Cd1—N4—C16	10.5 (5)	Cd1—O4—C21—C22	−172.2 (7)
O1—Cd1—N4—C16	88.2 (6)	O5—C21—C22—F1'	111.4 (14)
N2—Cd1—N4—C16	−86.4 (6)	O4—C21—C22—F1'	−64.4 (15)
N1—Cd1—N4—C16	−43.3 (7)	O5—C21—C22—F2'	−118.5 (13)
O2—Cd1—N4—C16	93.2 (6)	O4—C21—C22—F2'	65.8 (14)
O4—Cd1—N4—C20	−7.4 (6)	O5—C21—C22—F3	57.0 (18)
N3—Cd1—N4—C20	−179.2 (7)	O4—C21—C22—F3	−118.7 (15)
O1—Cd1—N4—C20	−101.5 (6)	O5—C21—C22—F2	−70.8 (17)
N2—Cd1—N4—C20	83.9 (6)	O4—C21—C22—F2	113.5 (14)
N1—Cd1—N4—C20	127.0 (6)	O5—C21—C22—F1	172.4 (13)
O2—Cd1—N4—C20	−96.5 (6)	O4—C21—C22—F1	−3.4 (16)
Cd1—O2—N5—O3	175.8 (8)	O5—C21—C22—F3'	−4.4 (15)
Cd1—O2—N5—O1	−4.4 (7)	O4—C21—C22—F3'	179.9 (10)

### Hydrogen-bond geometry (Å, °)

**Table:**

*D*—H···*A*	*D*—H	H···*A*	*D*···*A*	*D*—H···*A*
C7—H7···O5^i^	0.93	2.44	3.160 (13)	134
C19—H19···O2^ii^	0.93	2.52	3.320 (11)	145
C13—H13···O3^iii^	0.93	2.43	3.287 (12)	152
C14—H14···O2^iv^	0.93	2.44	3.294 (11)	152

Symmetry codes: (i) −*x*+2, *y*−1/2, −*z*+1/2; (ii) −*x*+1, *y*−1/2, −*z*+1/2; (iii) *x*, −*y*+3/2, *z*−1/2; (iv) −*x*+1, −*y*+1, −*z*.
